# A dataset of micro-scale tomograms of unidirectional glass fiber/epoxy and carbon fiber/epoxy composites acquired via synchrotron computed tomography during *in-situ* tensile loading

**DOI:** 10.1016/j.dib.2020.106672

**Published:** 2020-12-19

**Authors:** Mahoor Mehdikhani, Christian Breite, Yentl Swolfs, Martine Wevers, Stepan V. Lomov, Larissa Gorbatikh

**Affiliations:** KU Leuven, Department of Materials Engineering, Kasteelpark Arenberg 44, 3001, Leuven, Belgium

**Keywords:** Fiber-reinforced composites, X-ray computed tomography, Synchrotron, In-situ loading, Microstructural analysis, Misalignment, Fiber breaks

## Abstract

We have performed synchrotron computed tomography on two different fiber-reinforced composites while they were being continuously *in-situ* loaded in 0° tension. One material is a glass/epoxy laminate and the other is a carbon/epoxy laminate. The voxel size is 1.1 µm, which allows clear recognition of the glass fibers, but not distinct individual carbon fibers. For each material, four loading steps are selected with approximately 0, 40, 73, and 95% of the failure load, and the 3D images of the four volumes from each material are overlaid. A volume of interest in the middle 0° ply is chosen and located in the 3D image of each loading step (Fig. 1). The cropped volumes of interest for each material are presented in this publication and are publicly available on *Mendeley Data*[1]. As examples of two frequently-used type of unidirectional fiber-reinforced composites, the presented data can be used for different microstructural analyses, including investigation of the 3D variability in fiber distribution and orientation, and their evolution during tensile loading. For example, we have performed fiber orientation analysis on this data, using our digital image correlation-based technique, in [2]. Moreover, real-time formation of fiber breaks with tensile loading can be investigated in the data.

## Specifications Table

**Table:** 

Subject	Ceramics and Composites
Specific subject area	Microstructural analysis of fiber-reinforced polymers via synchrotron computed tomography
Type of data	Image
How data were acquired	Synchrotron X-ray computed tomography (TOMCAT beamline at SLS): Source: 2.9-Tesla superbending magnet; Photon source size (h, v) = 140 µm, 45 µm (FWHM); Photon source divergence (tailored by aperture) (h, v) = 2 mrad (top-hat), 0.6 mrad (FWHM) Scintillator: LuAG:Ce; Thickness = 20 µm Microscope: High resolution white-beam microscope (Optique Peter) M Plan Apo 10x; Magnification = 10.0; Numerical Aperture = 0.28; Focal length = 200 mm Detector: GigaFRoST (PSI in-house); Pixel size = 11.0 µm; Sensor size (h × v) = 2016 × 1716 px² (300 px in the vertical direction were turned off due to a scratch on the scintillator) Reconstruction: with an absorption-based algorithm provided by SLS
Data format	Raw – 8 bit TIFF format Filtered (non-local means and ring artifact) – 8 bit TIFF format
Parameters for data collection	Propagation distance = 60 mm Beam energy = 20 kV Nr. of projections = 1000 (2500 for one of the images) Exposure time = 9 ms (30 ms for one of the images) Voxel size = 1.1 μm Magnification = 10 ×
Description of data collection	Double-edge notched specimen of a glass/epoxy and a carbon/epoxy laminate were scanned via synchrotron radiation while being continuously loaded *in-situ* tension. For each material, captured volumes of four selected loading steps are overlaid, and a volume of interest is cropped from each 3D image. To reduce the image noise, 3D non-local means filter is applied. Both unfiltered and filtered image datasets are provided here.
Data source location	Institution: Swiss Light Source (SLS) at Paul Scherrer Institute (PSI) City/Town/Region: Villigen/Brugg Country: Switzerland Latitude and longitude (and GPS coordinates, if possible) for collected samples/data: 47°32′04.5″N 8°13′17.2″E
Data accessibility	Repository name: Mendeley Data Data identification number: DOI:10.17632/jn63c55y32.1 Direct URL to data: https://data.mendeley.com/datasets/jn63c55y32/1
Related research article	Mehdikhani M, C Breite, Y Swolfs, M Wevers, SV Lomov, L Gorbatikh, Combining digital image correlation with X-ray computed tomography for characterization of fiber orientation in unidirectional composites, Composites Part A. In Press.

## Value of the Data

• The tomograms provide 3D microstructural data of two very common Fiber-Reinforced Composites (FRCs), which allow detailed investigation of (evolution of) real-life microstructural variability that is extremely useful for simulation of FRCs accounting for their variability.
• The presented data can be useful for materials scientists, mechanical engineers, and computer vision experts, who study (experimentally or computationally) the processing, variability, and thermo-mechanical behavior of FRCs.
• The data can enhance the algorithms for random generation of 3D fibers, used to create representative volume elements for modeling of thermo-mechanical behavior of FRCs. Moreover, the data not only provides information on 3D microstructural variability (e.g. in fiber distribution and orientation), but also serves as a test case for future techniques that aim at measuring 3D variability in FRCs.
• The data allows investigation of the evolution of microstructure with tensile loading, which includes, for example, the change in fiber distribution/alignment and the formation of fiber breaks.

## 1 Data Description

The data presented in this article, and available on *Mendeley Data* [1], is in the form of 3D images, which are provided as image stacks. Each stack consists of 2D image slices that are in 8-bit TIFF format. In total, sixteen 3D images are supplied: eight raw (unfiltered) and eight filtered. Each of these datasets consists of four 3D images corresponding to the four loading steps of the glass/epoxy Volume Of Interest (VOI) and four images of loading steps of the carbon/epoxy VOI. Therefore, the data file includes two subfolders: Raw and Filtered (non-local means), each consisting of two subfolders: Carbon-epoxy and Glass-epoxy, each including four subfolders: Step0, …, Step3. For clarification, the data structure is presented in Table 1.

**Table 1:** Structure of the presented data in the current article – available on *Mendeley Data*[1].

	Raw	Filtered (non-local means and ring artifact)
Glass-epoxy	Step0	Step0
	Step1	Step1
	Step2	Step2
	Step3	Step3
Carbon-epoxy	Step0	Step0
	Step1	Step1
	Step2	Step2
	Step3	Step3

## 2 Experimental Design, Materials and Methods

Two composite laminates were produced: a cross-ply [90_9_/0_9_]_s_ glass/epoxy with PPG *Hybon 2026* glass fibers, and a cross-ply [90_4_/0_5_]_s_ carbon/epoxy laminate with *Mitsubishi 34–700WD* carbon. The nominal fiber diameter is 15 µm for the glass and 7 µm for the carbon. According to [4], the measured average diameters are 13.4 µm and 6.5 µm, respectively. Prepregs were made with *736LT* epoxy by *North Thin Ply Technology*. The laminates were manually stacked and cured at 70 °C for 60 min and at 120 °C temperature and 0.5 MPa pressure for 45 min inside an autoclave, while a vacuum of −0.7 bar was maintained. The thickness of the cured glass/epoxy laminate is 0.9 mm and of the carbon/epoxy laminate is 1 mm.

**Fig. 1:**
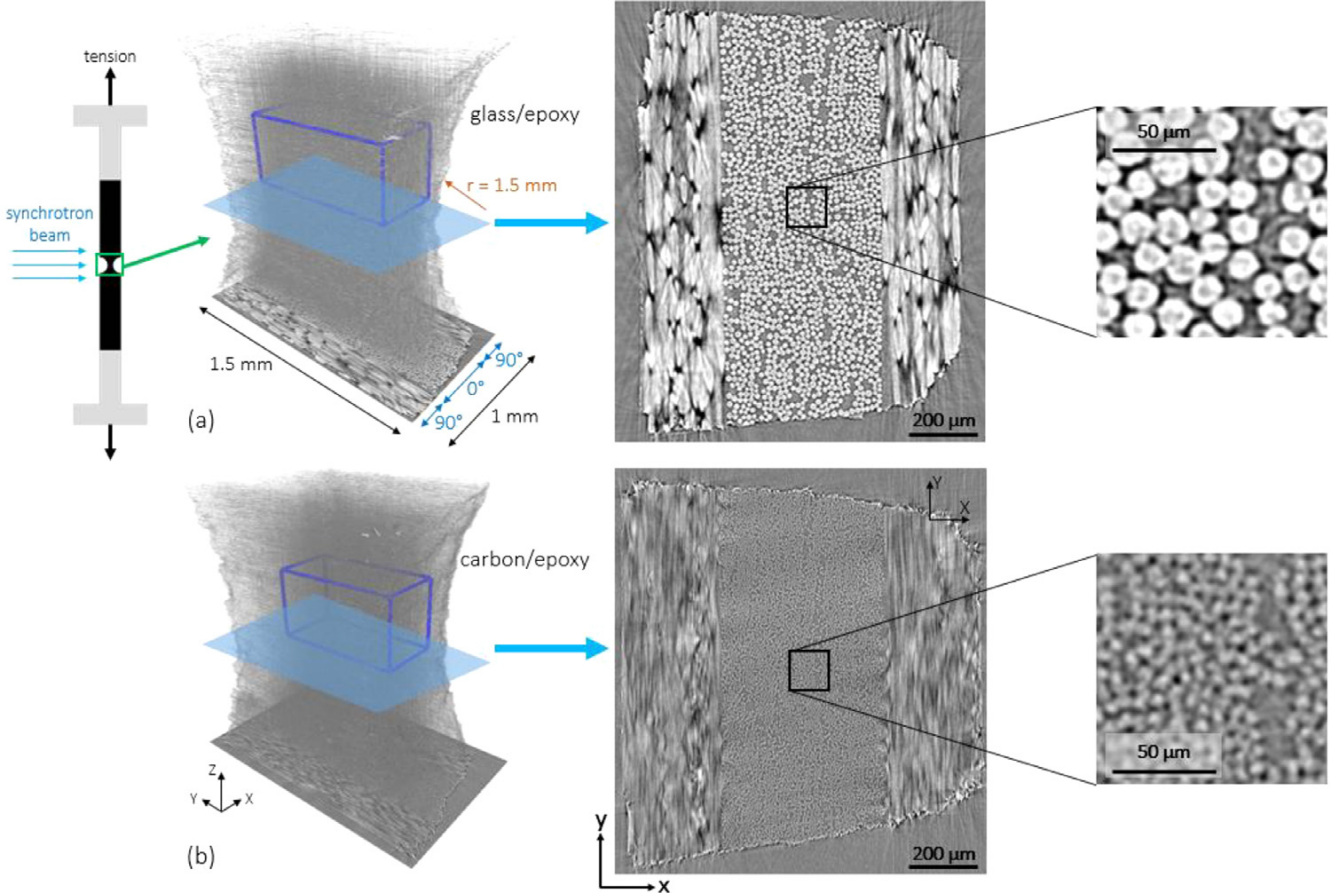
3D rendering representation and an example *yx* slice acquired via synchrotron scanning of (a) the glass/epoxy and (b) carbon/epoxy cross-ply laminates – the blue boxes correspond to the volume of interests presented in this article – figure taken from [2].

For *in-situ* scanning during tension, miniature tensile specimens with a special geometry are needed. A double-edge notched specimen, as shown in Fig. 1, was cut from each laminate using waterjet machining, similar to the specimens in [3]. The notch section area is ∼ 1 × 1 mm^2^. Each specimen was loaded in 0° tension with a deformation rate of ∼ 1.5 μm/s using a displacement-controlled loading rig provided by *INSA Lyon*. The specimen was scanned during the continuous loading with *in-situ* synchrotron X-ray. The tests were performed at the *TOMCAT* beamline of the *Swiss Light Source* (*SLS*) in Villigen, Switzerland. The continuous scans were made with a beam energy of 20 kV, a propagation distance of 60 mm, and 1000 projections with an exposure time of 9 ms. The first (undeformed) scan of the glass/epoxy material was made, before the loading started, with 2500 projections of 30-ms, while the first scan of the carbon/epoxy material was made with the abovementioned settings when the loading just started. The voxel size was 1.1 μm, and the magnification was 10 × . The reconstruction of the projection into 3D volumes was done using a filtered back-projection algorithm provided by *SLS*.

For each material, around 60 volumes were acquired during the continuous loading, before the final failure. This was feasible thanks to the very short scan time of only 9 s, while it takes multiple hours to make a comparable scan on lab tomography equipment. The continuous loading eliminates the effect of stress relaxation during scanning as highlighted in [5]. The volumes were slightly (<10°) rotated, using bicubic interpolation in MATLAB, so that the image coordinate system equals the laminate coordinate system: *z*- and *y*-axes are aligned respectively with the nominal 0° and 90° lamina directions, and *x*-axis is normal to the laminate plane (Fig. 1). For each material, four of the volumes were selected, corresponding to the steps with ∼ 0, 40, 73, and 95% of the failure load, and presented in the current study. Using the *Avizo 2019.3* software, the volumes of each material were registered (overlaid) on each other via 3D rigid transform and with “Normalized Mutual Information” and resampled with “Standard” (linear) interpolation. This eliminates the rigid body motion and rotation occurred during loading and allows easily locating the VOIs.

A VOI of 420 × 1030 × 450 μm^3^ (*x, y, z*) for the glass/epoxy and of 390 × 830 × 440 μm^3^ (*x, y, z*) for the carbon/epoxy material was chosen in their middle 0° plies (the blue box in Fig. 1a and b, respectively) and cropped from the larger volumes. The VOIs represent unidirectional plies and cover most of the unnotched volume for each material. The VOI of the glass/epoxy material contains around 1100 fibers. However, the number of fibers in the carbon/epoxy VOI cannot be exactly determined due to the low diameter-to-voxel size ratio (using a fiber volume fraction of 48%, the number of fibers is estimated to be ∼ 4500). A “Ring Artifact Removal” filter was applied to the VOIs *Avizo 2019.3*. Except the first (undeformed) volume of the glass/epoxy material that was scanned with more and longer projections, the other volumes were rather noisy. Hence, a 3D “non-local means” filter was applied to those VOIs in *Avizo* with a search window of 10 px and a local neighborhood of 3 px.

## Ethics Statement

This work has not involved any use of human subjects and animal experiments.

## Declaration of Competing Interest

The authors declare that they have no known competing financial interests or personal relationships which have, or could be perceived to have, influenced the work reported in this article.

## Data Availability

A dataset of micro-scale tomograms of unidirectional glass fiber/epoxy and carbon fiber/epoxy composites acquired via synchrotron computed tomography during in-situ tensile loading (Original data) (Mendeley Data).
